# Supplementary figures and images for: Patient-mix, programmatic characteristics, retention and predictors of attrition among patients starting antiretroviral therapy (ART) before and after the implementation of HIV “Treat All” in Zimbabwe
Source: PLoS One. 2020 Oct 19;15(10):e0240865. doi: 10.1371/journal.pone.0240865 (PMC7571688; doi:10.1371/journal.pone.0240865)

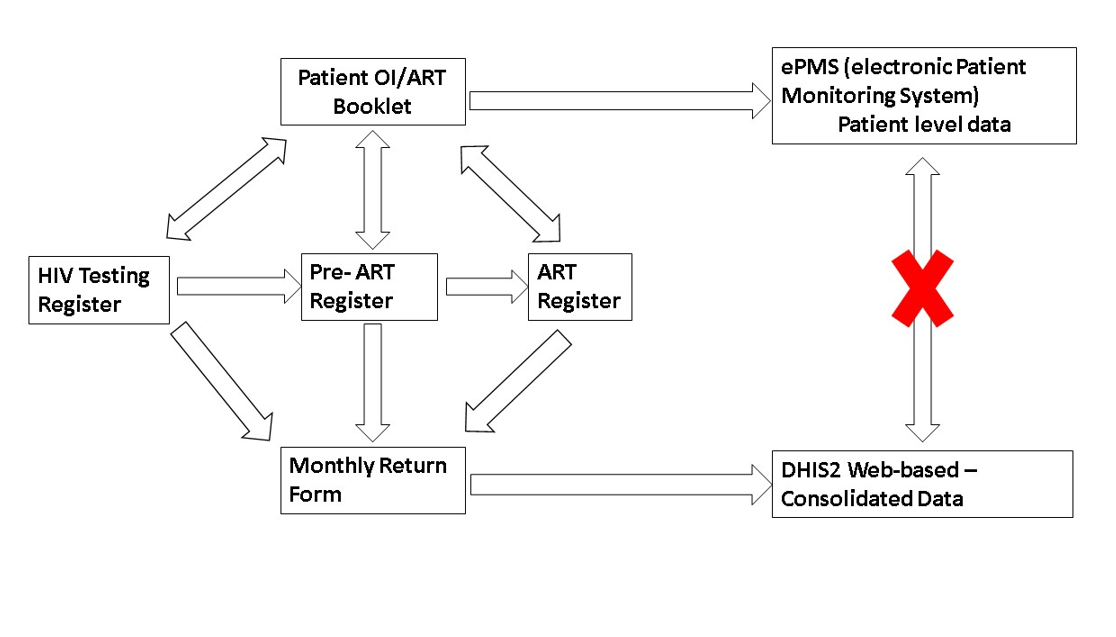

Supplement: S1 Fig — (TIF) [file pone.0240865.s001.tif]
