# Supplementary material for: Patient-mix, programmatic characteristics, retention and predictors of attrition among patients starting antiretroviral therapy (ART) before and after the implementation of HIV “Treat All” in Zimbabwe
Source: PLoS One. 2020 Oct 19;15(10):e0240865. doi: 10.1371/journal.pone.0240865 (PMC7571688; doi:10.1371/journal.pone.0240865)
Supplement: S1 Table — (DOCX) [file pone.0240865.s002.docx]

**S1 Table: Outcomes, follow time and time from testing to ART initiation for patients who started antiretroviral therapy before and after the implementation of HIV “Treat All” in the 9 pilots districts in Zimbabwe.**

|  | **Before “Treat All”** |  | **After “Treat All”** |  | **Combined** |  |
| --- | --- | --- | --- | --- | --- | --- |
|  |  |  |  |  |  |  |
| **Outcome** | **Frequency** | **(%)** | **Frequency** | **(%)** | **Frequency** | **(%)** |
| Active | 1458 | 83.9 | 1747 | 85.3 | 3205.0 | 84.6 |
| Transferred | 138 | 7.9 | 137 | 6.7 | 275.0 | 7.3 |
| Attrition | 142 | 8.2 | 165 | 8.1 | 307.0 | 8.1 |
| **Total** | **1738** | **100** | **2049** | 100.0 | **3787** | **100** |
|  | | | | | | |
| **Attrition** |  |  |  |  |  |  |
| LTFU | 102 | 71.8 | 112 | 67.9 | 214 | 69.7 |
| Stopped ART | 14 | 9.9 | 38 | 23.0 | 52 | 16.9 |
| Died | 26 | 18.3 | 15 | 9.1 | 41 | 13.4 |
| **Total** | **142** | **100.0** | **165** | **100.0** | **307** | **100,0** |
|  | | | | | | |
| **Follow up time (months)** |  |  |  |  |  |  |
| Maximum | 32.9 |  | 23.8 |  | 32.9 |  |
| Median (IQR) | 19.7(16.1-28.1) |  | 16 (8.9 - 19.7) |  | 18.1(11.2 -21.5) |  |
|  |  |  |  |  |  |  |
|  | | | | | | |
| **Testing to ART initiation (Days)** |  |  |  |  |  |  |
| Observations | 1516 |  | 1840 |  |  |  |
| Q_1_ | 5.0 |  | 0.0 |  |  |  |
| Median | 21.0 |  | 1.0 |  |  |  |
| Q_3_ | 94.5 |  | 24.0 |  |  |  |
|  |  |  |  |  |  |  |
| *ART: antiretroviral therapy, LFTU: Lost to follow-up, IQR: interquartile range, Q_1_: 25^th^ percentile, Q_3_: 75^th^ percentile* | | | | | | |
