# Supplementary material for: Patient-mix, programmatic characteristics, retention and predictors of attrition among patients starting antiretroviral therapy (ART) before and after the implementation of HIV “Treat All” in Zimbabwe
Source: PLoS One. 2020 Oct 19;15(10):e0240865. doi: 10.1371/journal.pone.0240865 (PMC7571688; doi:10.1371/journal.pone.0240865)
Supplement: S2 Table — (DOCX) [file pone.0240865.s003.docx]

**S2 Table: Attrition for patients who started antiretroviral therapy before and after the implementation of HIV “Treat All” in the 9 pilots districts in Zimbabwe**

| **Variable** | **Categories** | **Total** | **Attrition^$^** | **Person-time years** | **Attrition^$^ (100PY)** |
| --- | --- | --- | --- | --- | --- |
| **Total** |  | **3787** | **307** | **5516.0** | **5.7** |
| Cohort | Before “Treat All” | 1738 | 142 | 3025.4 | 4.7 |
|  | After “Treat All” | 2049 | 165 | 2490.6 | 6.6 |
| Sex | Female | 2351 | 182 | 2101.2 | 5.3 |
|  | Male | 1436 | 125 | 3414.8 | 5.9 |
| Age group | Adults | 2783 | 235 | 4005.1 | 6.0 |
|  | Children | 151 | 10 | 230.2 | 4.3 |
|  | Adolescents and young adults | 345 | 37 | 476.9 | 7.8 |
|  | Elderly | 508 | 25 | 803.8 | 3.1 |
| HIV testing modality | Voluntary | 1270 | 106 | 1841.8 | 5.8 |
|  | Antenatal | 549 | 62 | 752.5 | 8.2 |
|  | Others | 1438 | 105 | 2120.9 | 5.0 |
|  | Missing | 530 | 34 | 800.8 | 4.2 |
| Baseline tuberculosis status | Negative screening | 3519 | 280 | 5124.7 | 5.5 |
|  | Presumptive tuberculosis | 31 | 5 | 40.4 | 12.4 |
|  | On tuberculosis treatment | 132 | 13 | 199.4 | 6.5 |
|  | Missing | 105 | 9 | 151.5 | 5.9 |
| WHO stage | I-III | 3738 | 302 | 5450.7 | 5.5 |
|  | IV | 31 | 5 | 46.1 | 10.6 |
|  | Missing | 18 | 0 | 19.2 | 0.0 |
| Functional status | Normal | 298 | 20 | 5036.6 | 5.7 |
|  | Impaired | 3488 | 287 | 478.0 | 4.2 |
|  | Missing | 1 | 0 | 1.4 | 0.0 |
| Level of care | District/provincial hospital | 1307 | 133 | 2073.8 | 5.6 |
|  | Primary health facility | 2480 | 174 | 3442.2 | 5.1 |
| Partner support | Not supported | 444 | 30 | 786.0 | 3.8 |
|  | Supported | 3343 | 277 | 4730.0 | 5.9 |
| Pregnant when starting ART | *No | 3241 | 238 | 4766.8 | 5.0 |
|  | Yes | 546 | 69 | 749.2 | 9.2 |
|  |  |  |  |  |  |
| *$ either stopped ART, death or LTFU,* **Includes non-pregnant women and men, PY: person-time years, ART: antiretroviral therapy, WHO: World Health Organisation, Children: 0-14 years, Adolescents and young adults: 15-24 years, Adults: 25 – 49 years, Elderly: +50 years* | | | | | |
