# Supplementary material for: Patient-mix, programmatic characteristics, retention and predictors of attrition among patients starting antiretroviral therapy (ART) before and after the implementation of HIV “Treat All” in Zimbabwe
Source: PLoS One. 2020 Oct 19;15(10):e0240865. doi: 10.1371/journal.pone.0240865 (PMC7571688; doi:10.1371/journal.pone.0240865)
Supplement: Attachment — Submitted filename: PONE-D-20-20707_VJG_review_28_07_20.docx [file pone.0240865.s006.docx]

**PLOS ONE**

### Patient and programmatic characteristics, retention and predictors of attrition among patients starting antiretroviral therapy (ART) before and after the implementation of HIV “Treat All” in Zimbabwe

--Manuscript Draft--

| **Manuscript Number:** | PONE-D-20-20707 |
| --- | --- |
| **Article Type:** | Research Article |
| **Full Title:** | Patient and programmatic characteristics, retention and predictors of attrition among patients starting antiretroviral therapy (ART) before and after the implementation of HIV “Treat All” in Zimbabwe |
| **Short Title:** | Comparison of attrition and risk factors before and after HIV “Treat All” implementation in Zimbabwe |
| **Corresponding Author:** | Richard Makurumidze Institute of Tropical Medicine Antwerpen, BELGIUM |
| **Keywords:** | HIV "Treat All", low resource setting, effectiveness, outcomes, retention, risk factors, Zimbabwe, Africa |
| **Abstract:** | Background  Since scale-up of the HIV “Treat All”, evidence on its real-world effect on known predictors of attrition (either death or lost to follow-up) is lacking. We conducted a retrospective study using Zimbabwe ART program data to assess the association between “Treat All” and, patient and programmatic characteristics, retention and predictors of attrition.  Methods  We used patient-level data from the electronic patient monitoring system (ePMS) from the nine districts which piloted “Treat All”. We compared patient and programme characteristics, retention and predictors of attrition (lost to follow-up, death or stopping ART) in two cohorts; before (April/May 2016) and after (January/February 2017) “Treat All”. Retention was estimated using survival analysis. Predictors of attrition were determined using a multivariable Cox regression model. Interactions were used to assess the change in predictors.  Results  We analysed 3787 patients, 1738 (45.9%) and 2049 (54.1%) started ART before and after “Treat All”, respectively. The proportion of men was higher after “Treat All” (39.4.% vs 36.2%, p=0.044). Same-day ART initiation was more frequent after “Treat All” (43.2% vs 16.4%; p<0.001) than before. Retention on ART was higher before “Treat All” (p<0.001). Among non-pregnant women and men, the adjusted hazard ratio (aHR) of attrition after “Treat All” compared to before “Treat All” was 1.73 (95%CI: 1.30  - 2.31). The observed hazard of attrition for women being pregnant at ART initiation decreased by 17% (aHR: 1.73*0.48 = 0.83). Being male (vs female; aHR: 1.45; 95%CI:  1.12 - 1.87) and WHO Stage IV (vs WHO Stage I- III; aHR: 2.89; 95%CI: 1.16 - 7.11) predicted attrition both before and after “Treat All” implementation.  Conclusion  Attrition was higher after “Treat All”; being male, WHO Stage 4, and pregnancy predicted attrition in both before and after Treat All. However, pregnancy became a less strong risk factor for attrition after “Treat All” implementation.” |
| **Order of Authors:** | Richard Makurumidze |
|  | Jozefien Buyze |
|  | Tom Decroo |
|  | Lutgarde Lynen |
|  | Madelon de Rooij |
|  | Trevor Mataranyika |
|  |  |

*Powered by Editorial Manager® and ProduXion Manager® from Aries Systems Corporation*

|  | Ngwarayi Sithole |
| --- | --- |
|  | Kudakwashe C Takarinda |
|  | Tsitsi Apollo |
|  | James Hakim |
|  | Wim Van Damme |
|  | Simbarashe Rusakaniko |
| **Additional Information:** |  |
| **Question** | **Response** |
| **Financial Disclosure**  Enter a financial disclosure statement that describes the sources of funding for the work included in this submission. Review the [submission guidelines](http://journals.plos.org/plosone/s/submission-guidelines#loc-financial-disclosure-statement) for detailed requirements. View published research articles from [*PLOS ONE*](http://journals.plos.org/plosone/) for specific examples.  This statement is required for submission and **will appear in the published article** if the submission is accepted. Please make sure it is accurate. | Richard Makurumidze receives a PhD scholarship grant from the Institute of Tropical Medicine, funded by the Belgian Development Cooperation (DGD). The funders had no role in study design, data collection and analysis, decision to publish, or preparation of the manuscript. |
| **Unfunded studies**  Enter: *The author(s) received no specific funding for this work.*  **Funded studies**  Enter a statement with the following details:   - Initials of the authors who received each award - Grant numbers awarded to each author - The full name of each funder - URL of each funder website - Did the sponsors or funders play any role in the study design, data collection and analysis, decision to publish, or preparation of the manuscript? - **NO** - Include this sentence at the end of your statement: *The funders had no role in study design, data collection and analysis, decision to publish, or preparation of the manuscript.* - **YES** - Specify the role(s) played. |  |
| * typeset |  |
| **Competing Interests**  Use the instructions below to enter a | The authors declare that they have no competing interests. |

*Powered by Editorial Manager® and ProduXion Manager® from Aries Systems Corporation*

| competing interest statement for this submission. On behalf of all authors, disclose any [competing interests](http://journals.plos.org/plosone/s/competing-interests) that could be perceived to bias this work—acknowledging all financial support and any other relevant financial or non- financial competing interests.  This statement **will appear in the published article** if the submission is accepted. Please make sure it is accurate. View published research articles from [*PLOS ONE*](http://journals.plos.org/plosone/) for specific examples. |  |
| --- | --- |
| **NO authors have competing interests**  Enter: *The authors have declared that no competing interests exist.*  **Authors with competing interests**  Enter competing interest details beginning with this statement:  *I have read the journal's policy and the authors of this manuscript have the following competing interests: [insert competing interests here]* |  |
| * typeset |  |
| **Ethics Statement**  Enter an ethics statement for this submission. This statement is required if the study involved:   - Human participants - Human specimens or tissue - Vertebrate animals or cephalopods - Vertebrate embryos or tissues - Field research   Write "N/A" if the submission does not require an ethics statement.  General guidance is provided below. Consult the [submission guidelines](http://journals.plos.org/plosone/s/submission-guidelines#loc-guidelines-for-specific-study-types) for detailed instructions. **Make sure that all information entered here is included in the** | This study was submitted for ethical review and approval to the Institutional Review Board (IRB-1257/18) of the Institute of Tropical Medicine, Antwerp, Belgium; University of Zimbabwe Joint Research Ethics Committee (JREC/239/18) and Medical Research Council of Zimbabwe (MRCZ/A/2410). In addition, permission to conduct the study was sought from the Ministry of Health and Child Care. The data were anonymised to maintain privacy and confidentiality. |

*Powered by Editorial Manager® and ProduXion Manager® from Aries Systems Corporation*

| **Methods section of the manuscript.** |  |
| --- | --- |
| **Format for specific study types**  **Human Subject Research (involving human participants and/or tissue)**   - Give the name of the institutional review board or ethics committee that approved the study - Include the approval number and/or a statement indicating approval of this research - Indicate the form of consent obtained (written/oral) or the reason that consent was not obtained (e.g. the data were analyzed anonymously)   **Animal Research (involving vertebrate animals, embryos or tissues)**   - Provide the name of the Institutional Animal Care and Use Committee (IACUC) or other relevant ethics board that reviewed the study protocol, and indicate whether they approved this research or granted a formal waiver of ethical approval - Include an approval number if one was obtained - If the study involved *non-human primates*, add *additional details* about animal welfare and steps taken to ameliorate suffering - If anesthesia, euthanasia, or any kind of animal sacrifice is part of the study, include briefly which substances and/or methods were applied   **Field Research**  Include the following details if this study involves the collection of plant, animal, or other materials from a natural setting:   - Field permit number - Name of the institution or relevant body that granted permission |  |
|  |  |
| **Data Availability**  Authors are required to make all data underlying the findings described fully available, without restriction, and from the time of publication. PLOS allows rare exceptions to address legal and ethical concerns. See the [PLOS Data Policy](http://journals.plos.org/plosone/s/data-availability) and [FAQ](http://journals.plos.org/plosone/s/data-availability#loc-faqs-for-data-policy) for detailed information. | No - some restrictions will apply |

*Powered by Editorial Manager® and ProduXion Manager® from Aries Systems Corporation*

| A Data Availability Statement describing where the data can be found is required at submission. Your answers to this question constitute the Data Availability Statement and **will be published in the article**, if accepted. |  |
| --- | --- |
| **Important:** Stating ‘data available on request from the author’ is not sufficient. If your data are only available upon request, select ‘No’ for the first question and explain your exceptional situation in the text box. |  |
| Do the authors confirm that all data underlying the findings described in their manuscript are fully available without restriction? |  |
| **Describe where the data may be found in full sentences. If you are copying our sample text, replace any instances of XXX with the appropriate details.**   - If the data are **held or will be held in a public repository**, include URLs, accession numbers or DOIs. If this information will only be available after acceptance, indicate this by ticking the box below. For example: *All XXX files are available from the XXX database (accession number(s) XXX, XXX.*). - If the data are all contained **within the manuscript and/or Supporting Information files**, enter the following: *All relevant data are within the manuscript and its Supporting Information files.* - If neither of these applies but you are able to provide **details of access elsewhere**, with or without limitations, please do so. For example:   *Data cannot be shared publicly because of [XXX]. Data are available from the XXX Institutional Data Access / Ethics Committee (contact via XXX) for researchers who meet the criteria for access to confidential data.*  *The data underlying the results* | The study was conducted with routinely collected program data of the Zimbabwe National ART Programme available in an electronic patient management system (ePMS) However, the data is not available on the public domain, and anyone interested in using the data for scientific or academic purpose should contact the Director of the AIDS and TB Program, Ministry of Health and Child Care, Government of Zimbabwe, 2nd Floor, Mukwati Building, Harare, Zimbabwe. Email: [atp.director@ymail.com](mailto:atp.director@ymail.com) |

*Powered by Editorial Manager® and ProduXion Manager® from Aries Systems Corporation*

| *presented in the study are available from (include the name of the third party and contact information or URL).*   - This text is appropriate if the data are owned by a third party and authors do not have permission to share the data.   * typeset |  |
| --- | --- |
| Additional data availability information: |  |

*Powered by Editorial Manager® and ProduXion Manager® from Aries Systems Corporation*

Manuscript	[Click here to access/download;Manuscript;Manuscript.docx](https://www.editorialmanager.com/pone/download.aspx?id=26947728&amp;guid=468a8996-889b-4927-9308-417fe224d5e5&amp;scheme=1)

### 1 Patient and programmatic characteristics, retention and predictors of

2 **attrition among patients starting antiretroviral therapy (ART) before and**
3 **after the implementation of HIV “Treat All” in Zimbabwe**
4 Richard Makurumidze ^1,2,3*^, Jozefien Buyze ^2^, Tom Decroo ^2,5^, Lutgarde Lynen ^2^,
5 Madelon de Rooij ^1^, Trevor Mataranyika ^4^, Ngwarayi Sithole ^4^, Kudakwashe C.
6 Takarinda ^4,6^, Tsitsi Apollo ^4^, James Hakim ^1^, Wim Van Damme ^2,3^, Simbarashe
7 Rusakaniko 1

8

9 1. College of Health Sciences, University of Zimbabwe, Harare, Zimbabwe
10 2. Institute of Tropical Medicine, Antwerp, Belgium
11 3. Gerontology, Faculty of Medicine & Pharmacy, Free University of Brussels
12 (VUB), Brussels, Belgium
13 4. AIDS & TB Unit, Ministry of Health & Child Care, Harare, Zimbabwe
14 5. Research Foundation of Flanders, Brussels, Belgium
15 6. International Union Against Tuberculosis and Lung Disease, Paris, France.

16

17	*** Corresponding Author:** Email: [rmakurumidze@ext.itg.be](mailto:rmakurumidze@ext.itg.be) (RM)

18

19

20

21

22

# 23 Abstract

## 24 Background

25 Since scale-up of the HIV “Treat All”, evidence on its real-world effect on known
26 predictors of attrition (either death or lost to follow-up) is lacking. We conducted a
27 retrospective study using Zimbabwe ART program data to assess the association
28 between “Treat All” and, patient and programmatic characteristics, retention and
29 predictors of attrition.

## 30 Methods

31 We used patient-level data from the electronic patient monitoring system (ePMS)
32 from the nine districts which piloted “Treat All”. We compared patient and
33 programme characteristics, retention and predictors of attrition (lost to follow-up,
34 death or stopping ART) in two cohorts; before (April/May 2016) and after
35 (January/February 2017) “Treat All”. Retention was estimated using survival analysis.
36 Predictors of attrition were determined using a multivariable Cox regression model.
37 Interactions were used to assess the change in predictors.

## 38 Results

39 We analysed 3787 patients, 1738 (45.9%) and 2049 (54.1%) started ART before and
40 after “Treat All”, respectively. The proportion of men was higher after “Treat All”
41 (39.4.% vs 36.2%, p=0.044). Same-day ART initiation was more frequent after “Treat
42 All” (43.2% vs 16.4%; p<0.001) than before. Retention on ART was higher before “Treat
43 All” (p<0.001). Among non-pregnant women and men, the adjusted hazard ratio (aHR)
44 of attrition after “Treat All” compared to before “Treat All” was 1.73 (95%CI: 1.30 -
45 2.31). The observed hazard of attrition for women being pregnant at ART initiation
46 decreased by 17% (aHR: 1.73*0.48 = 0.83). Being male (vs female; aHR: 1.45; 95%CI:

47	1.12 - 1.87) and WHO Stage IV (vs WHO Stage I- III; aHR: 2.89; 95%CI: 1.16 - 7.11)

48	predicted attrition both before and after “Treat All” implementation.

49

## 50 Conclusion

51 Attrition was higher after “Treat All”; being male, WHO Stage 4, and pregnancy
52 predicted attrition in both before and after Treat All. However, pregnancy became a
53 less strong risk factor for attrition after “Treat All” implementation.”

54

55 **Keywords:** HIV “Treat All”, low resource setting, effectiveness, outcomes,
56 retention, risk factors, Zimbabwe, Africa
57 **Short title:** Comparison of attrition and risk factors before and after HIV “Treat All”
58 implementation in Zimbabwe

59

60

61

62

63

64

65

66

# 67 Introduction

68

69 The highest number of people living with HIV (PLHIV) originate from the East and
70 Southern African (ESA) region. However, this region has not yet met the Joint United
71 Nations Programme for HIV/AIDS (UNAIDS) 90-90-90 targets, which were launched in
72 2014 to achieve epidemic control by 2020 [1]. By the end of 2018 in the ESA region,
73 85% [95% confidence interval (CI); 75-95%] of PLHIV knew their HIV status, with 67%
74 of those diagnosed on antiretroviral therapy (ART) and only 58%
75 of those on ART achieving sustained viral load suppression [2]. To
76 close the gap, by the end of 2018, 93% of low- and middle-income countries and 100%
77 of those designated as Fast Track countries had adopted the HIV “Treat All” policy [3].

78

79 Several clinical trials have shown the positive effects of “Treat All” on ART initiation,
80 linkage to care, virologic suppression, and retention [4,5]. Multiple studies assessed
81 the real-world effects of “Treat All” on ART initiation, linkage, virologic suppression,
82 and retention. However, these studies have reported inconsistent results [6–13].
83 None of the previous studies assessed if predictors for attrition changed since the
84 transition to “Treat All”. There is also still lack of evidence on the effect of “Treat All”
85 on the patient mix, i.e. change in the uptake of ART services among previously
86 underserved groups outside of trial settings. In Zimbabwe, as in many other African
87 countries, men and adolescents and young adults remain underserved [14–17].
88 Whether since the scale-up of “Treat All” the uptake of HIV care services, have
89 increased in favour of these previously underserved subgroups is unknown. Moreover,
90 it is not fully understood how programmatic procedures related to the provision of
91 HIV care across the cascade have been adapted and influence the outcomes.

92

93 Since July 2016 Zimbabwe started “Treat All” implementation. We used program data
94 from those districts that piloted “Treat All” before it was expanded to the rest of
95 Zimbabwe. We assessed the association between “Treat All” and, patient and
96 programme characteristics, retention and predictors of attrition by comparing the ART
97 cohorts enrolled during two periods before and after implementation of the “Treat
98 All” policy. This is the first study to assess the performance of the ART programme in
99 Zimbabwe since the start of HIV “Treat All” policy implementation and a follow-up on
100 previous national studies on ART outcomes in the country.

## 102 Methods and Materials

103 **Study design**
104 A retrospective cohort study using routinely collected individual patient data was
105 conducted.

## 106 Study setting

107 Zimbabwe has a generalised HIV epidemic with an estimated number of PLHIV of 1.4
108 million [18]. The country has made significant progress towards achieving the UNAIDS
109 90-90-90 targets: 76.8% of PLHIV are aware of their HIV status; of that 88.4 % were on
110 ART and among those on ART, 85.3% were virally suppressed [15]. The study used data
111 from the nine districts (Chipinge, Bulilima, Gwanda, Harare, Mangwe, Makoni,
112 Mazowe, Mutasa and Mutare) that piloted “Treat All”. Zimbabwe is geographically
113 divided into ten provinces and 63 districts. The nine pilot districts were purposively
114 selected from 4 provinces supported by the United States Presidential Emergency Plan
115 for AIDS Relief (PEPFAR).

## 117 Implementation of “Treat All” in the pilot districts

118 The pilot started in July 2016. By August of the same year, all nine pilot districts were
119 implementing “Treat All”. Before the start of “Treat All”, health care workers were
120 trained on the rationale of Treat All, clinical assessment of opportunistic infections,
121 ART initiation, adherence counselling and patient follow-up, tracking and call back of
122 clients who were not ART because of the previous eligibility criteria and quantification
123 of ART commodities. One-day meetings were conducted in all districts to sensitise
124 community stakeholders, including traditional and political leaders, community-based
125 organisations and networks of PLHIV on the new “Treat All” criteria and its benefits.
126 Four technical working groups were established to monitor progress on four different
127 themes: training and capacity building, linkage and referral of clients, learning and
128 documentation and communication. PEPFAR funded the pilot through its two main
129 implementing partners (International Training Education and Centre for Health - I-
130 TECH and Organisation for Public Health Intervention Development - OPHID). The
131 Ministry of Health and Child Care provided the necessary comprehensive support
132 during all stages of the pilot.

## 133 Study population

134 Health facilities were included if they used an HIV programme electronic medical
135 record, i.e. the electronic patient monitoring system (ePMS), which was mainly used
136 in high-volume sites. Of 385 health facilities in the nine pilot districts, 131 (30%) had
137 ePMS. Of 131 health facilities with ePMS,	72 had updated data in the national
138 database at the end of December 2018 and were included in the study. The before
139 “Treat All” cohort and the after “Treat All” cohort included all patients who started
140 ART between April-May 2016 and January -February 2017, respectively.

## 142 Data sources and study procedures

143 The patient’s medical data were first captured in a paper-based medical record
144 (Patient ART/Opportunistic Infection Booklet). The data were then entered in the
145 ePMS by either health care workers or data entry clerks. Every month health facilities
146 submit an encrypted back-up of the ePMS data to the district where all data are
147 consolidated. The consolidated district-level data is then submitted to the national
148 level, where it is further consolidated into one national database. Data for this study
149 were extracted from the national database and cleaned (synchronised and
150 deduplicated) before analysis (S1 Figure ).

## 152 Study variables

153 The	variables	extracted	for	analysis	included	patient	and	programmatic
154 characteristics. Patient characteristics included demographics (age and sex), and
155 baseline clinical parameters at ART initiation (functional status, WHO Stage,
156 pregnancy, tuberculosis status). Programmatic characteristics included HIV testing
157 modality, level of care, ART regimen and time from HIV testing to ART initiation. The
158 patient follow-up status (active on treatment, lost to follow-up (LTFU), dead,
159 transferred or stopped ART) and date were also collected.

## 161 Data analysis

162 The data were analysed using Stata version 16.0 (Stata Corp, College Station, Texas,
163 USA) [19]. Descriptive statistics (frequencies and proportions or medians and
164 quartiles) were used to describe and compare patient and programmatic
165 characteristics (before and after “Treat All”). The Chi-square or Fisher’s exact test were
166 used to compare the distribution for the categorical patient and programmatic
167 characteristics before “Treat All” with the distribution after “Treat All”. The Wilcoxon
168 rank-sum test and t-test were used for skewed and symmetrical continuous variables,
169 respectively. The adverse outcome event was attrition, which included those who
170 died, those LTFU and those who stopped ART. LTFU were those whose last recorded
171 clinic visit date, or pharmacy pill pick-up date, was ≥180 days before the date of data
172 extraction from the ePMS. Patients were considered active on ART when their last
173 recorded clinic visit date or pharmacy pill pick-up date was <180 days before the date
174 of data extraction from the ePMS. We calculated the proportion of LTFU patients who
175 only visited the health facility once on the day of ART initiation by dividing the number
176 LTFU patients who only visited the health facility once on the day of ART initiation with
177 the total number initiated on ART in the cohort. In the time-to-event analysis, patients
178 classified as active on ART were censored on the date of their most recently recorded
179 clinic visit or pharmacy pick-up while those who transferred out were censored on the
180 date of transfer out. The survival time was calculated as the time from the date of ART
181 initiation to the date of censoring or attrition. Kaplan Meier survival	curves and
182 statistics were used to estimate retention at 6, 12 and 24 months. The log-rank test
183 was used to compare survival curves for different strata.
185 In the bivariate and multivariate analysis, we included variables with less than 30%
186 missingness. Missing data of included variables were managed through imputation.
187 The data were assumed to be missing at random. Multiple imputation was conducted
188 using multiple chains equations, and twenty imputed datasets were created and used
189 for analysis. Cox proportional hazard models with a frailty variable for health facility
190 were used to identify predictors of attrition. Proportional hazard assumptions were
191 tested by comparing observed with predicted survival curves and log-log plots. The
192 cohort variable (before “Treat All”, after “Treat All”) was included in all bivariate and
193 multivariate models. A hierarchical approach was employed. All variables associated
194 with P-value <0.1 in the bivariate analysis were included in the multivariate model.
195 Sex was maintained in the model because of clinical and programmatic relevance.
196 Stepwise backward elimination was used until all variables in the model had a p-value
197 < 0.05. To be able to evaluate whether the hazard of significant predictors changed
198 between the before and after “Treat All” cohort we looked at the interactions
199 between the significant variables and our primary exposure variable (i.e. cohort). They
200 were assessed and added to the model, using the same hierarchical approach and
201 stepwise backward elimination approach described above.

## 202 Ethical (and regulatory) review

203 This study was submitted for ethical review and approval to the Institutional Review
204 Board (IRB-1257/18) of the Institute of Tropical Medicine, Antwerp, Belgium;
205 University of Zimbabwe Joint Research Ethics Committee (JREC/239/18) and Medical
206 Research Council of Zimbabwe (MRCZ/A/2410). In addition, permission to conduct the
207 study was sought from the Ministry of Health and Child Care. The data were
208 anonymised to maintain privacy and confidentiality.

# 211 Results

#### 212 a) Study participants

213 We analysed 3787 patients; 1738 (45.9%) from the before and 2049 (54.1%) from the
214 after “Treat All” cohorts.
217 **b) Association	between	“Treat	All”	and	patient	and	programmatic**
218 **characteristics**
219 In the before and after “Treat All” cohort median age was 37 [interquartile range (IQR),
220 30-44] and 36 (IQR, 29-43) years, respectively. The proportion of men among those
221 who started ART was significantly higher after “Treat All” compared to before “Treat
222 All” cohort (39.4% vs 36.2%, p =0.044). There was no significant increase in the
223 proportion of adolescents among those who started ART. The proportion of
224 asymptomatic patients (WHO Stage 1) was higher in the after “Treat All” cohort (42.5%

226	vs 26.1%, p <0.001) (Table 1).

227

228 The proportion of patients tested for HIV through voluntary counselling and testing
229 among all tested was higher after “Treat All” (37.7% vs 28.6%, p <0.001) while the
230 proportion tested in antenatal care services decreased (11.9% vs 17.6%, p <0.001).
231 There was no significant change in the distribution of patients started on ART by the
232 level of care. The median number of days between HIV diagnosis and ART initiation
233 before and after “Treat All” were 21 (IQR, 5-95) and 1 (IQR, 0-24) days, respectively
234 (S1 Table). The proportion of patients who started ART on the same day increased

235	(43.2% vs 16.4%; p <0.001) (Table 2)

#### 236 Table 1: Comparison of baseline patient characteristics starting ART before (April-May 2016) and after (January-February 2017) the

237 **implementation of HIV “Treat All” in 9 pilots districts in Zimbabwe**

| **Variables** | **Categories** | **Before “Treat All”** | | **After “Treat All”** | | **p-valuea** |
| --- | --- | --- | --- | --- | --- | --- |
|  |  | **n** | **(%)** | **n** | **(%)** |  |
| Total |  | 1738 | (100) | 2049 | (100) |  |
| Sex | Male | 629 | (36.2) | 807 | (39.4) | **0.044** |
|  | Female | 1109 | (63.8) | 1242 | (60.6) |  |
| Median age (Q1 – Q3) | | 37 (30-44) |  | 36 (29-43) |  | **0.008b** |
| Age groups | Children | 67 | (3.9) | 84 | (4.1) |  |
|  | Adolescents and young adults | 145 | (8.3) | 200 | (9.8) | 0.092 |
|  | Adults | 1270 | (73.1) | 1513 | (73.8) |  |
|  | Elderly | 256 | (14.7) | 252 | (12.3) |  |
| Functional Status | Bedridden | 1 | (0.1) | 3 | (0.1) | 0.220 |
|  | Ambulatory | 148 | (8.5) | 146 | (7.1) |  |
|  | Working | 1588 | (91.4) | 1900 | (92.7) |  |
|  | Missing | 1 | (0.1) | 0 | (0.0) |  |
| WHO Stage | WHO Clinical Stage 1 | 454 | (26.1) | 871 | (42.5) | **<0.001** |
|  | WHO Clinical Stage 2 | 772 | (44.4) | 804 | (39.2) |  |
|  | WHO Clinical Stage 3 | 480 | (27.6) | 357 | (17.4) |  |
|  | WHO Clinical Stage 4 | 19 | (1.1) | 12 | (0.6) |  |
|  | Missing | 13 | (0.7) | 5 | (0.2) |  |
| Pregnancy at ART initiation | Yes | 302 | (17.4) | 244 | (11.9) | **<0.001** |
|  | cNo | 1436 | (82.6) | 1805 | (88.1) |  |
| TB Status at ART Initiation | Negative Screening | 1611 | (92.7) | 1908 | (93.1) | **0.023** |

|  | TB Presumptive | 15 | (0.9) | 16 | (0.8) |  |
| --- | --- | --- | --- | --- | --- | --- |
|  | On TB Treatment | 74 | (4.3) | 58 | (2.8) |  |
|  | Missing | 38 | (2.2) | 67 | (3.3) |  |
| *aChi-square test*, ^b^Wilcoxon *rank-sum test, ^c^Includes non-pregnant women and men,* ART: *antiretroviral therapy, TB: tuberculosis, IQR: interquartile range, WHO: World Health Organisation, Children: 0-14 years, Adolescents and young adults: 15-24 years, Adults: 25 – 49 years, Elderly: +50 years* | | | | | | |

242 **Table 2: Comparison of programmatic characteristics before and after the implementation of HIV “Treat All” in 9 pilots districts in**
243 **Zimbabwe**

| **Variables** | **Categories** | **Before “Treat All”** |  | **After “Treat All”** |  | **ap-value** |
| --- | --- | --- | --- | --- | --- | --- |
|  |  | **n** | **(%)** | **n** | **(%)** |  |
| Total |  | 1738 | (100) | 2049 | (100) |  |
| HIV testing modality | Antenatal | 306 | (17.6) | 243 | (11.9) | **<0.001** |
|  | OI, TB & Illness | 580 | (33.4) | 634 | (30.9) |  |
|  | Occupational | 42 | (2.4) | 67 | (3.3) |  |
|  | Voluntary | 497 | (28.6) | 773 | (37.7) |  |
|  | Others | 63 | (3.6) | 52 | (2.5) |  |
|  | Missing | 250 | (14.4) | 280 | (13.7) |  |
| Level of care | Clinic | 1143 | (65.8) | 1337 | (65.3) | 0.421 |
|  | District/Mission Hospital | 460 | (26.5) | 571 | (27.9) |  |
|  | Provincial Hospital | 135 | (7.8) | 141 | (6.9) |  |
| ART Regimen | TDF/3TC/EFV | 1646 | (94.7) | 1962 | (95.8) | 0.221 |
|  | AZT/3TC/NVP(Children) | 43 | (2.5) | 41 | (2.0) |  |

|  | AZT/3TC/LPV/r(Children) | 19 | (1.1) | 17 | (0.8) |  |
| --- | --- | --- | --- | --- | --- | --- |
|  | Others | 39 | (2,2) | 29 | (1.4) |  |
| Time between HIV diagnosis and ART initiaton (days) | Same day | 285 | (16.4) | 885 | (43.2) | **<0.001** |
|  | 1 - 14 days | 342 | (19.7) | 404 | (19.7) |  |
|  | 15 - 90 days | 501 | (28.8) | 283 | (13.8) |  |
|  | > 90 days | 388 | (22.3) | 268 | (13.1) |  |
|  | Missing | 222 | (12.8) | 209 | (10.2) |  |
| *aChi-square test, ART: Antiretroviral therapy, OI: opportunistic infection, TB: tuberculosis, TDF: tenofovir, AZT: zidovudine, 3TC: lamivudine, NVP: nevirapine, EFV: efavirenz, LPV/r: lopinavir/ritonavir* | | | | | | |

247 **c) Association between retention and “Treat All”**
248 The maximum follow-up time for the before and after “Treat All” cohorts were 32.9 and 23.8 months while the median was 19.7 (IQR, 6.1 –
249 28.1) and 16.0 (IQR, 8.9 – 19.7) months respectively. The total attrition for the before “Treat All” cohort was 142/1738 (8,2%; 9.9% stopped ART,
250 71.8% LTFU and 18.3% died) and after “Treat All” cohort was 165/2049 (8,1%; 23.0% stopped ART, 67.9% LTFU and 9% died) (S1 Table). The
251 proportion of LTFU patients who only visited the health facility once on the day of ART initiation among all patients initiated on ART increased
252 after “Treat All” (2.1% vs 0.5%, p <0.001). Comparing the two cohorts, the 6 and 12 months retention for the before and after “Treat All” were

253	98.5% (95%CI; 97.8– 99.0) and 95.1% (95%CI; 94.0 – 96.1) and, 97.0% (95%CI; 96.2 – 97.7) and 94.1 % (95%CI; 92.9 – 95.1) respectively (Table

254 3). Retention during the first 12 months of treatment was higher before “Treat All” (log-rank, p<0.001) (Figure 2).

#### 255 Table 3: Combined, before and after HIV “Treat All” retention in care of patients

256 **who started antiretroviral therapy in 9 pilot districts in Zimbabwe**

| **Cohort** | **Months since ART**  **initiation** | **Retention (%)** | **95% Confidence**  **Interval** |
| --- | --- | --- | --- |
| Combined | 6 | (97.7) | (97.1 – 98.1) |
|  | 12 | (94.5) | (93.7– 95.2) |
|  | 24 | (88.1) | (86.6 – 89.5) |
| Before “Treat  All” | 6 | (98.5) | (97.8– 99.0) |
|  | 12 | (95.1) | (94.0 – 96.1) |
|  | 24 | (90.4) | (88.6 – 92.0) |
| After “Treat All” | 6 | (97.0) | (96.2 – 97.7) |
|  | 12 | (94.1) | (92.9 – 95.1) |
|  |  |  |  |
| *After “Treat All”: no patients had a follow-up of 24 months* | | | |

260 **Figure 2: Comparison of the retention at 12 months for patients who started**
261 **before and after the implementation of HIV “Treat All” in the 9 pilots districts in**
262 **Zimbabwe**
265 **d)	Predictors of attrition before and after the implementation of “Treat All”**
266 Among non-pregnant women and men, the adjusted hazard ratio (aHR) of attrition
267 after “Treat All” compared to before “Treat All” was 1.73 (95%CI: 1.30 - 2.31). The
268 observed hazard of attrition for women being pregnant at ART initiation decreased by
269 17% (aHR: 1.73*0.48 = 0.83) since “Treat All”. Being male (vs female; aHR: 1.45; 95%CI:

270	1.12 - 1.87) and WHO Stage IV (vs WHO Stage I- III; aHR: 2.89; 95%CI: 1.16 - 7.11)

271	predicted attrition both before and after “Treat All” implementation.

272

273 The hazard ratio of attrition comparing pregnant women to non-pregnant women was
274 significantly different (aHR: 0.48; 95%CI: 0.27- 0.85) after “Treat All” compared to
275 before “Treat All”: the hazard ratio was 3.47 (95%CI: 2.36 - 5.11) before “Treat All”
276 and 1.67 (95%CI: ) after “Treat All.”. The association of gender and WHO stage with
277 attrition was not significantly different after “Treat All” compared to before “Treat
278 All”.

#### 281 Table 4. Bivariate and multivariate analysis of factors associated with attrition for patients who started antiretroviral therapy before and

282 **after the implementation of HIV “Treat All” in the 9 pilots districts in Zimbabwe (Multiple imputation, N= 3787)**

| **Variable** | **Categories** | **HR** | **p-value** | **(95%CI)** | **aHR** | **p-value** | **(95%CI)** |
| --- | --- | --- | --- | --- | --- | --- | --- |
|  |  |  |  |  |  |  |  |
| Cohort | Before “Treat All” | 1 |  |  |  |  |  |
|  | After “Treat All” | **1.40** | **0.009** | **(1.09 - 1.80)** | **1.73** | **<0.001** | **(1.30 - 2.31)** |
| Sex | Female | 1 |  |  |  |  |  |
|  | Male | 1.12 | 0.336 | (0.89 - 1.41) | **1.45** | **0.005** | **(1.12- 1.87)** |
| Age group | Adults | 1 |  |  |  |  |  |
|  | Children | 0.80 | **0.010*** | (0.42 - 1.52) |  |  |  |
|  | Adolescents and  young adults | 1.30 |  | (0.91 - 1.85) |  |  |  |
|  | Elderly | **0.55** |  | **(0.37 - 0.84)** |  |  |  |
| HIV testing  modality | Voluntary | 1 |  |  |  |  |  |
|  | Antenatal | **1.52** | **0.002*** | **(1.10 - 2.11)** |  |  |  |
|  | Others | 0.84 |  | (0.64 - 1.12) |  |  |  |
| Baseline  tuberculosis status | Negative screening | 1 |  |  |  |  |  |
|  | Presumptive  tuberculosis | 2.05 | 0.233* | (0.83 - 5.06) |  |  |  |
|  | On tuberculosis  treatment | 1.23 |  | (0.70 - 2.16) |  |  |  |
| WHO stage | I-III | 1 |  |  |  |  |  |
|  | IV | **2.52** | **0.0450** | **(1.02- 6.21)** | **2.89** | **0.0220** | **(1.16- 7.11)** |
| Functional status | Normal | 1 |  |  |  |  |  |
|  | Impaired | 1.06 | 0.830 | (0.62 - 1.83) |  |  |  |

| Level of care | District/provincial  hospital | 1 |  |  |  |  |  |
| --- | --- | --- | --- | --- | --- | --- | --- |
|  | Primary health  facility | 0.81 | 0.448 | (0.47 - 1.39) |  |  |  |
| Partner support | Not supported | 1 |  |  |  |  |  |
|  | Supported | 1.31 | 0.432 | (0.66 - 2.61) |  |  |  |
| Pregnant when  starting ART | a No | 1 |  |  |  |  |  |
|  | Yes | **2.08** | **<0.001** | **(1.58 - 2.75)** | **3.47** | **<0.001** | **(2.36- 5.11)** |
| Pregnancy##Cohort | Not confirmed#After  “Treat All” |  |  |  | 1 |  |  |
|  | Confirmed#After  “Treat All” |  |  |  | **0.48** | **0.0120** | **(0.27- 0.85)** |
| **Overall p-value ^a^ Includes non-pregnant women and men, HR: hazard ratio, aHR: adjusted hazard ratio, CI: confidence interval, WHO: World Health Organisation, ART: antiretroviral therapy, #: interaction, Children: 0-14 years, Adolescents and young adults: 15-24 years, Adults: 25 – 49 years, Elderly: +50 years* | | | | | | | |

289 **Discussion**
290 **Summary of main findings**
291 This is the first study to assess the performance of the ART programme in Zimbabwe
292 since the scale-up of “Treat All” policy. The study is also a follow-up on previous
293 national studies on ART outcomes in the country. “Treat All” implementation resulted
294 in lower retention and a higher risk of attrition. While the proportion of men among
295 those who started on ART during “Treat All” increased, the proportion of adolescents
296 and young adults among those starting ART did not significantly change between the
297 two periods. After “Treat All” more patients initiated ART on the same day as the HIV
298 diagnosis was made. Being male, WHO Stage 4, and pregnancy predicted attrition in
299 both before and after Treat All. However, pregnancy became a less strong risk factor

for attrition after “Treat All” implementation.

#### 300 Association between “Treat All” and patient and programmatic characteristics

301 In our study, we found the proportion of men in those starting ART under “Treat All”
302 was higher than before. A programmatic report from Lesotho also reported an
303 increase in the proportion of men among those starting ART under “Treat All” [20].
304 These findings are however contrary to an assessment which was conducted in 6 sub-
305 Saharan African countries to assess the effects of HIV “Treat All” on rapid ART
306 initiation, showing that men remain at risk of not initiating ART [21]. We believe that
307 in our study the reason for a higher proportion of men among those initiating ART
308 might have been due to demand creation, HIV testing and ART initiation strategies
309 that were employed as part of “Treat All” implementation such as moonlight, index
310 case testing and HIV self-testing [22–24]. The increase in patients testing through
311 voluntary HIV testing services, as shown by this study, might also have benefited men.
313 We found that “Treat All” implementation did not increase the proportion of
314 adolescents and young adults among those starting ART. To date, studies on whether
315 HIV “Treat All” increases rapid ART initiation among adolescents are inconclusive.
316 Positive effects have been shown in some studies, while others have shown that they
317 are still at risk of not initiating ART [21,25–27]. To our knowledge, no evidence-based
318 strategies are currently being implemented in Zimbabwe on a broader scale under
319 “Treat All” to promote ART initiation among adolescents and young adults. Strategies
320 to improve ART initiation among adolescents which include multidisciplinary and
321 adolescent‐friendly HIV services together with peer counselling and support, should
322 be tested [28,29]. In our study, we found that more patients are now being initiated
323 on ART on the same day. In clinical trials, same-day ART initiation has shown either no
324 difference or a positive effect on retention [30–33]. Few studies having been done to
325 assess the real-world effects of same-day ART initiation [34], a call for further
326 research. Whether patients are adequately prepared, both psychological and clinically
327 on the same day remains a critical question.
329 To date, the few studies assessing the real-life effects of “Treat All” have shown
330 inconsistent findings regarding its effect on retention. Some have shown patients
331 starting under “Treat All” likely to do better[11,12], others worse [6,7] while others
332 showed no difference [8–10]. In our study, patients who started ART during HIV “Treat
333 All” had lower retention and a higher risk of attrition. This collided with findings from
334 our previous evaluation that patients starting ART at a higher CD4 cut-off might be at
335 risk of attrition [35]. Patients started on ART under “Treat All”, possibly have low-risk
336 perception due to the absence of symptoms and might be less motivated to adhere to
337 lifelong daily ART. [36,37]. The other reason might be during the implementation of
338 “Treat All” early on; more focus has been on ART initiation, including same-day ART
339 initiation. The health care workers were then unable to prepare and provide adequate
340 counselling on the importance of adhering to life-long ART to these asymptomatic
341 patients who might not appreciate its benefits [38]. This is supported by our finding
342 were there was a slight increase of the proportion of LTFU patients who only visited
343 the health facility once on the day of ART initiation among all patients initiated on ART
344 after Treat All. Another study conducted in Eswatini also showed similar findings where
345 the percentage of patients who never returned after the first ART visit doubled (from
346 3 to 6%) in the “Treat All” period [10]. Several factors can explain the conflicting
347 findings in studies looking at retention during “Treat All”, including different lengths
348 of the follow-up period, settings, methods, study population and definition of
349 outcomes. A systematic review and meta-analysis are needed to synthesise and
350 consolidate the evidence.
352 The decrease in retention under “Treat All” has also been confirmed by routine
353 programme reports from the national ART programme. This, therefore, calls for the
354 country to develop and implement innovative evidence-based strategies that have
355 been shown to improve retention. The process has already started with differentiated
356 ART delivery models (Community ART refills groups-CARGS, adherence clubs, fast
357 track and outreach) currently being scaled-up. By the end of 2018, around 30% of
358 stable adolescents and adults on ART were receiving ART through at least one
359 differentiated ART delivery model [39]. Systems to track and trace defaulters have also
360 been put in place. Despite systems to track defaulters, LTFU continues to be a
361 challenge. LTFU accounts for most of the attrition. This is thought to have also an
362 administrative reason [35], and the MoHCC and the supporting implementing partner
363 have since instituted a National ART Census Survey to be able to get correct estimates
364 of the number of patients on ART. The results are yet to become available in the public
365 domain.

#### 367 Predictors of attrition before and after the implementation of “Treat All”

368 In our study pregnancy became a less strong risk factor for attrition since “Treat All”
369 implementation, which is a positive development. We hypothesise that the reduction
370 might be due to the expansion of “Treat All” initiative to the rest of PLHIV. For a long
371 period, “Treat All” has been specifically focused on the subgroup of HIV positive
372 pregnant women and delivered as Option B+, which might have created some
373 “stigma” [40,41]. Now, this is applied to all patients; this label might have been
374 removed. Current efforts to retain pregnant and breastfeeding women on ART which
375 include the integration of services, family-centred approaches, and the use of	lay
376 healthcare providers which include mentor mothers should be optimised and
377 continued [42,43]. Documentation and follow-up of HIV positive pregnant women
378 with complications referred from lower to higher-level health facilities (maternal
379 waiting homes) should also be improved so that these referrals are not treated as
380 LTFU.
382 In the current study, adolescents and young adults had comparable retention with
383 adults contrary to other studies. The finding is contrary to other programmatic HIV
384 “Treat All” studies to date [6,7,9–11]. Zimbabwe has employed strategies to retain
385 adolescents in care, mainly through its home-grown Community adolescent’s
386 treatment supporters (CATS) model. Evidence has shown the CATS model to be
387 effective in retaining adolescents in HIV care [44]. To date, the model has been scaled
388 up to most of the districts. Resources to fully implement the CATS model throughout
389 the country should be mobilised.
391 We found men to be at risk of attrition under “Treat All”, a finding similar to other
392 studies [7,9]. Differentiated service delivery strategies that have been shown to retain
393 men in ART care under HIV “Treat All” which includes tailored awareness on the health
394 benefits of early ART start, accelerated linkage to care, decreasing logistical barriers
395 to HIV care, patient-centred approach, flexible clinic hours and tracking and tracing of
396 those missing appointments should be strengthened, maintained and optimised [45].

#### 398 Strength and limitations

399 Compared to other studies, our study sample size was large and representative. The
400 pilot districts were distributed across all the regions of the country. The study was the
401 first to assess the performance of the ART programme since the start of HIV “Treat
402 All” policy implementation and allowed us to test and confirm findings from our
403 previous evaluation.
404 However, our study had limitations. We evaluated the effect of HIV “Treat All”,
405 considering it as one intervention, though it has several components across the
406 cascade of care. We were unable to disentangle these components and assess their
407 individual effects on attrition. Our study was based on health facilities with ePMS,
408 which is only available in high volume health facilities, and this might affect the
409 generalisability. However, sites with ePMS contain most patients on ART in the
410 country. We could also not assess re-engagement in care since the ePMS database
411 only reports the most recent status of the patient with no record of previous
412 disengagements. Because we used routine electronic programme data collected
413 retrospectively, we had to deal with missing data. Data on weight, height, CD4 and
414 viral load were more than 50% missing, so could not be assessed as a confounder. To
415 assess the effect of disease progression on attrition, we then used WHO Staging and
416 functional status as proxies in our model. Most of the attrition in our study was
417 explained by LTFU. Some of these patients reported as LTFU might be still alive and
418 on ART (unreported self-transfers), stopped ART or unreported deaths [46].
420 Because of missing data, we were also unable to compare the viral load suppression
421 rates between the before and after HIV “Treat All” cohorts. Unfortunately, it was
422 impossible to solve this by merging ePMS and laboratory data, due to missing or
423 inconsistent format of the unique patient identifier between the various testing
424 laboratories. Strategies to improve data quality and making sure the existing
425 electronic data collection systems are interoperable need urgent attention. This is an
426 observational study looking into an association of individual patient and programme
427 characteristics and retention in care. We are aware that lumping patients from all
428 facilities together may have caused a loss of information on specific health facility-
429 level factors that influence retention, and that could not be studied.

#### 431 Future research

432 Considering inconsistency of evidence in the programmatic effects of “Treat All”, there
433 is a need for more research in the domain. From our field experience, we have noticed
434 the implementation of “Treat All” encompasses many aspects across the cascade of
435 care. The package includes innovative HIV testing and linkage to care strategies,
436 clinical and psychological readiness assessment, retesting before ART initiation, same-
437 day ART initiation, follow- up and retention in care strategies. On top of the HIV
438 cascade of care issues; there are also health systems challenges anticipated from the
439 abrupt increase in the number of patients now being followed-up in care. Further
440 research, both quantitative and qualitative, should explore the influence of all these
441 factors on “Treat All” implementation to optimise it. Men continue to be at risk under
442 “Treat All”; further research is needed on how ART delivery can be streamlined to
443 retain them in care.	Most studies have focused on patient-level factors on ART
444 outcomes. This, therefore, calls for studies looking into the impact of health facility-
445 level characteristics on ART outcomes since performance might vary across facilities.
446 Finally, as more studies looking into the real-world effects of “Treat All” surface, there
447 is a need for systematic reviews and meta-analyses summarising outcomes across the
448 cascade of care and evaluation of strategies to optimise “Treat All” implementation.

#### 450 Conclusion

451 In conclusion, our study found that since the implementation of “Treat All” the
452 retention in care was lower and the risk of attrition was higher. Male sex, advanced
453 HIV disease and pregnancy are risk factors for attrition both before and during the
454 implementation of HIV “Treat All”. However, after the implementation of “Treat All”
455 pregnancy became a less strong risk factor. Strategies to retain men and to improve
456 ART initiation among adolescents and young adults should be prioritised. Patients with
457 advanced disease (WHO Stage IV) continued to be at risk of attrition. Further research
458 should explore how different components of HIV “Treat All” implementation (HIV
459 testing, linkage, same-day ART initiation, clinical and psychological preparation) and
460 health system/facility issues have a bearing on patient outcomes.

# 462 Acknowledgements

463 The authors would like to thank the following organisations for support during the
464 study - Ministry of Health and Child Care (MoHCC); the University of Zimbabwe,
465 College of Health Sciences (UZCHS), Department of Community Medicine; Institute of
466 Tropical Medicine (ITM) in Antwerp, Belgium; the University of Zimbabwe, Zimbabwe
467 AIDS Prevention Project (ZAPP-UZ) and PEPFAR Zimbabwe and its main implementing
468 partners, I-TECH and OPHID for supporting the HIV “Treat All” pilot. The authors are
469 grateful to the team that assisted in ePMS data management prior to analysis. We also
470 want to various stakeholders and implementing partners who have been supporting
471 the MoHCC towards improving the MoHCC AIDS and TB Program data quality through
472 various initiatives, including supporting the ePMS. Of note is the Global Fund for AIDS,
473 Tuberculosis, and Malaria (GFATM) which supported MoHCC in setting up the ePMS.
474 Finally, we thank front line health workers and PLHIV on ART whose records were
475 used.

# 476 Authors Contributions

| **Author** | **Contribution** |
| --- | --- |
| Richard Makurumidze | Conceptualisation, Data curation, Formal analysis, Methodology, Investigation, Validation, Writing – original draft preparation, Project administration |
| Jozefien Buyze | Conceptualisation, Data curation, Formal analysis, Methodology, Validation, Writing – reviewing & editing |
| Tom Decroo | Conceptualisation, Methodology, Writing – review & editing |
| Lutgarde Lynen | Conceptualisation, Methodology, Supervision, Writing – review & editing, Resources, Funding Acquisition |
| Madelon de Rooij | Conceptualisation, Methodology, Writing – review & editing |
| Trevor Mataranyika | Conceptualisation, Data Curation, Methodology, Writing  – review & editing |
| Ngwarayi Sithole | Conceptualisation, Methodology, Writing – review & editing |
| Kudakwashe C. Takarinda | Conceptualisation, Methodology, Writing – review & editing |
| Tsitsi Apollo | Conceptualisation, Methodology, Writing – review & editing |
| James Hakim | Conceptualisation, Methodology, Writing – review & editing |
| Wim Van Damme | Conceptualisation, Methodology, Writing – review & editing |
| Simbarashe Rusakaniko | Conceptualisation, Methodology, Supervision, Writing – review & editing |

478 **Declarations**

## 479 Data Availability

480 The study was conducted with routinely collected program data of the Zimbabwe
481 National ART Programme available in an electronic patient management system
482 (ePMS) However, the data is not available on the public domain, and anyone
483 interested in using the data for scientific or academic purpose should contact the
484 Director of the AIDS and TB Program, Ministry of Health and Child Care, Government
485 of	Zimbabwe,	2nd	Floor,	Mukwati	Building,	Harare,	Zimbabwe.	Email:
486 [atp.director@ymail.com](mailto:atp.director@ymail.com)

## 488 Funding

489 Richard Makurumidze receives a PhD scholarship grant from the Institute of Tropical
490 Medicine, funded by the Belgian Development Cooperation (DGD). The funders had
491 no role in study design, data collection and analysis, decision to publish, or preparation
492 of the manuscript.

## 494 Competing Interests

495 The authors declare that they have no competing interests.

# 498 References

499 1.	UN Joint Programme on HIV/AIDS (UNAIDS). 90-90-90 An ambitious treatment
500 target to help end the AIDS epidemic. United Nations. 2014.
501 2.	The Joint United Nations Programme on HIV/AIDS (UNAIDS). UNAIDS Data.

506	2019.

507	3.	Brazier E, Maruri F, Duda SN, Tymejczyk O, Wester CW, Somi G, et al.

508	Implementation of “Treat‐all” at adult care and treatment sites in the Global

509	IeDEA Consortium: results from the Site Assessment Survey. J Int AIDS Soc.

510	2019;22. doi:10.1002/jia2.25331

511	4.	Perriat D, Balzer L, Hayes R, Lockman S, Walsh F, Ayles H, et al. Comparative

512	assessment of five trials of universal HIV testing and treatment in sub-Saharan

513	Africa. J Int AIDS Soc. 2018;21: e25048. doi:10.1002/jia2.25048

514	5.	Havlir D, Lockman S, Ayles H, Larmarange J, Chamie G, Gaolathe T, et al. What

515	do the Universal Test and Treat trials tell us about the path to HIV epidemic

516	control? J Int AIDS Soc. 2020;23: 1–7. doi:10.1002/jia2.25455

517	6.	Stafford KA, Odafe SF, Lo J, Ibrahim R, Ehoche A, Niyang M, et al. Evaluation of

518	the clinical outcomes of the Test and Treat strategy to implement Treat All in

519	Nigeria: Results from the Nigeria Multi-Center ART Study. Torpey K, editor.

520	PLoS One. 2019;14: e0218555. doi:10.1371/journal.pone.0218555

521	7.	Hirasen K, Fox MP, Hendrickson CJ, Sineke T, Onoya D. HIV Treatment

522	Outcomes Among Patients Initiated on Antiretroviral Therapy Pre and Post-

523	Universal Test and Treat Guidelines in South Africa. Ther Clin Risk Manag.

524	2020;Volume 16: 169–180. doi:10.2147/TCRM.S227290

525 8.	Ross J, Sinayobye J d. A, Yotebieng M, Hoover DR, Shi Q, Ribakare M, et al.
526 Early outcomes after implementation of treat all in Rwanda: an interrupted
527 time series study. J Int AIDS Soc. 2019;22: e25279. doi:10.1002/jia2.25279
528 9.	Dorward J, Sookrajh Y, Gate K, Khubone T, Mtshaka N, Mlisana K, et al. HIV
529 treatment outcomes among people with initiation CD4 counts >500 cells/µL
530 after implementation of Treat All in South African public clinics: a
531 retrospective cohort study. J Int AIDS Soc. 2020;23: 1–7.

532	doi:10.1002/jia2.25479

533	10.	Kerschberger B, Schomaker M, Jobanputra K, Kabore SM, Teck R, Mabhena E,

534	et al. HIV programmatic outcomes following implementation of the ‘Treat‐All’

535	policy in a public sector setting in Eswatini: a prospective cohort study. J Int

536	AIDS Soc. 2020;23: 1–15. doi:10.1002/jia2.25458

537	11.	Alhaj M, Amberbir A, Singogo E, Banda V, van Lettow M, Matengeni A, et al.

538	Retention on antiretroviral therapy during Universal Test and Treat

539	implementation in Zomba district, Malawi: a retrospective cohort study. J Int

540	AIDS Soc. 2019;22: e25239. doi:10.1002/jia2.25239

541	12.	Girum T, Yasin F, Wasie A, Shumbej T, Bekele F, Zeleke B. The effect of

542	“universal test and treat” program on HIV treatment outcomes and patient

543	survival among a cohort of adults taking antiretroviral treatment (ART) in low

544	income settings of Gurage zone, South Ethiopia. AIDS Res Ther. 2020;17: 19.

545	doi:10.1186/s12981-020-00274-3

546	13.	Kabogo J, Muniu E, Wamunyokoli F, Musoke R, Songok E. Evidence of reduced

547	treatment adherence among HIV infected paediatric and adolescent

548	populations in Nairobi at the onset of the UNAIDS Universal Test and Treat

549	Program. BMC Res Notes. 2018;11: 134. doi:10.1186/s13104-018-3205-0

550	14.	Simms V, Dauya E, Dakshina S, Bandason T, McHugh G, Munyati S, et al.

551	Community burden of undiagnosed HIV infection among adolescents in

552	Zimbabwe following primary healthcare-based provider-initiated HIV testing

553	and counselling: A cross-sectional survey. PLoS Med. 2017;14: 1–15.

554	doi:10.1371/journal.pmed.1002360

555	15.	Ministry of Health and Child Care Zimbabwe (MoHCC). Zimbabwe Population-

556	Based HIV Impact Assessment (ZIMPHIA) 2015-2016 - Final Report. 2019.

557	16.	Camlin CS, Ssemmondo E, Chamie G, Ayadi AM El, Kwarisiima D, Sang N, et al.

558	Men “ missing ” from population-based HIV testing : insights from qualitative

559	research. AIDS Care. 2016;0121. doi:10.1080/09540121.2016.1164806

560	17.	Katirayi L, Chadambuka A, Muchedzi A, Ahimbisibwe A, Musarandega R,

561	Woelk G, et al. Echoes of old HIV paradigms: Reassessing the problem of

562	engaging men in HIV testing and treatment through women’s perspectives.

563	Reprod Health. 2017;14: 1–13. doi:10.1186/s12978-017-0387-1

564	18.	Ministry of Health and Child Care(MoHCC). HIV Grant Global Fund report –

565	Semester 4 (July-December 2019). Harare, Zimbabwe; 2019.

566	19.	StataCorp. Stata Statistical Software: Release 16. Coll Station TX StataCorp

567	LLC. 2019;

568 20.	Schwitters AM. Preliminary Results After Implementation of a Universal
569 Treatment Program (Test and Start) for Persons Living with HIV Infection —
570 Lesotho, October 2015–February 2017. CDC, Morbidity and Mortality Weekly
571 Report. 2017.
572 21.	Tymejczyk O, Brazier E, Yiannoutsos CT, Vinikoor M, van Lettow M, Nalugoda
573 F, et al. Changes in rapid HIV treatment initiation after national “treat all”
574 policy adoption in 6 sub-Saharan African countries: Regression discontinuity
575 analysis. Newell M-L, editor. PLOS Med. 2019;16: e1002822.

576	doi:10.1371/journal.pmed.1002822

577 22.	Euphemia Sibanda, Melissa Neuman, Mary Tumushime, Karin Hatzold,
578 Constancia Watadzaushe, Elizabeth L. Corbett FC. Linkage to care after HIV
579 self-testing in Zimbabwe: a cluster-randomised trial. Conf RETROVIRUSES
580 OPPORTUNISTIC Infect Boston, Massachusetts March 8–11. 2018; Available:
581 <http://brunswickhealth.com.au/self-testing-kits-static.html>
582 23.	Mahachi N, Muchedzi A, Tafuma TA, Mawora P, Kariuki L, Semo B, et al.
583 Sustained high case‐finding through index testing and partner notification
584 services: experiences from three provinces in Zimbabwe. J Int AIDS Soc.

585	2019;22: 23–30. doi:10.1002/jia2.25321

586 24.	Richard Makurumidze, Jonga Aaron, Bochner BM. Reaching Men With HIV
587 Testing Services: Is Moonlight Testing Part of the Answer? Poster Present 19th
588 Int Conf AIDS STIs Africa (ICASA); Abidjan, Côte d’Ivoire. 2017;
589 25.	Tymejczyk O, Brazier E, Wools-Kaloustian K, Davies M-A, DiLorenzo M,
590 Edmonds A, et al. Impact of universal antiretroviral treatment eligibility on
591 rapid treatment initiation among young adolescents with HIV in sub-Saharan

592	Africa. J Infect Dis. 2019;10027: 1–9. doi:10.1093/infdis/jiz547

593 26.	Chihana ML, Huerga H, Van Cutsem G, Ellman T, Wanjala S, Masiku C, et al.
594 Impact of “test and treat” recommendations on eligibility for antiretroviral
595 treatment: Cross sectional population survey data from three high HIV
596 prevalence countries. PLoS One. 2018; doi:10.1371/journal.pone.0207656
597 27.	Kerschberger B, Jobanputra K, Schomaker M, Kabore SM, Teck R, Mabhena E,
598 et al. Feasibility of antiretroviral therapy initiation under the treat‐all policy
599 under routine conditions: a prospective cohort study from Eswatini. J Int AIDS

600	Soc. 2019;22. doi:10.1002/jia2.25401

601	28.	MacPherson P, Munthali C, Ferguson J, Armstrong A, Kranzer K, Ferrand RA, et

602	al. Service delivery interventions to improve adolescents’ linkage, retention

603	and adherence to antiretroviral therapy and HIV care. Trop Med Int Heal.

604	2015;20: 1015–1032. doi:10.1111/tmi.12517

605	29.	Shaw S, Amico KR. Antiretroviral Therapy Adherence Enhancing Interventions

606	for Adolescents and Young Adults 13–24 Years of Age. JAIDS J Acquir Immune

607	Defic Syndr. 2016;72: 387–399. doi:10.1097/QAI.0000000000000977

608	30.	Labhardt ND, Ringera I, Lejone TI, Klimkait T, Muhairwe J, Amstutz A, et al.

609	Effect of offering same-day ART vs usual health facility referral during home-

610	based HIV testing on linkage to care and viral suppression among adults with

611	HIV in Lesotho: The CASCADE randomised clinical trial. JAMA - J Am Med

612	Assoc. 2018;319: 1103–1112. doi:10.1001/jama.2018.1818

613	31.	Koenig SP, Dorvil N, Dévieux JG, Hedt-Gauthier BL, Riviere C, Faustin M, et al.

614	Same-day HIV testing with initiation of antiretroviral therapy versus standard

615	care for persons living with HIV: A randomised unblinded trial. PLoS Med.

616	2017;14: 1–15. doi:10.1371/journal.pmed.1002357

617	32.	Rosen S, Maskew M, Fox MP, Nyoni C, Mongwenyana C, Malete G, et al.

618	Initiating Antiretroviral Therapy for HIV at a Patient’s First Clinic Visit: The

619	RapIT Randomized Controlled Trial. Binagwaho A, editor. PLOS Med. 2016;13:

620	e1002015. doi:10.1371/journal.pmed.1002015

621	33.	Rosen S, Maskew M, Larson BA, Brennan AT, Tsikhutsu I, Fox MP, et al.

622	Simplified clinical algorithm for identifying patients eligible for same-day HIV

623	treatment initiation (SLATE): Results from an individually randomised trial in

624	South Africa and Kenya. PLoS Med. 2019;16: 1–20.

625	doi:10.1371/journal.pmed.1002912

626	34.	Lilian RR, Rees K, McIntyre JA, Struthers HE, Peters RPH. Same-day

627	antiretroviral therapy initiation for HIV-infected adults in South Africa:

628	Analysis of routine data. PLoS One. 2020;15: 1–13.

629	doi:10.1371/journal.pone.0227572

630	35.	Makurumidze R, Mutasa-Apollo T, Decroo T, Choto RC, Takarinda KC,

631	Dzangare J, et al. Retention and predictors of attrition among patients who

632	started antiretroviral therapy in Zimbabwe’s national antiretroviral therapy

633	programme between 2012 and 2015. PLoS One. 2020;15.

634	doi:10.1371/journal.pone.0222309

635	36.	Stafford KA, Odafe SF, Lo J, Ibrahim R, Ehoche A, Niyang M, et al. Evaluation of

636	the clinical outcomes of the Test and Treat strategy to implement Treat All in

637	Nigeria: Results from the Nigeria Multi-Center ART Study. Torpey K, editor.

638	PLoS One. 2019;14: e0218555. doi:10.1371/journal.pone.0218555

639	37.	Charurat M, Oyegunle M, Benjamin R, Habib A, Eze E, Ele P, et al. Patient

640	Retention and Adherence to Antiretrovirals in a Large Antiretroviral Therapy

641	Program in Nigeria: A Longitudinal Analysis for Risk Factors. Myer L, editor.

642	PLoS One. 2010;5: e10584. doi:10.1371/journal.pone.0010584

643	38.	Bock P, James A, Nikuze A, Peton N, Sabapathy K, Mills E, et al. Baseline CD4

644	Count and Adherence to Antiretroviral Therapy. Weiser SD, editor. JAIDS J

645	Acquir Immune Defic Syndr. 2016;73: 514–521.

646	doi:10.1097/QAI.0000000000001092

647 39.	United States of America President’s Emergency Plan for AIDS Relief (PEPFAR).
648 Country Operational Plan 2019 Strategic Direction Summary. 2019; 80.
649 Available: <http://www.pepfar.gov/documents/organization/250290.pdf>
650 40.	Chanda BC, Likwa RN, Zgambo J, Tembo L, Jacobs C. Acceptability of option B+
651 among HIV positive women receiving antenatal and postnatal care services in
652 selected health centre’s in Lusaka. BMC Pregnancy Childbirth. 2018;18: 510.

653	doi:10.1186/s12884-018-2142-1

654	41.	Knettel BA, Cichowitz C, Ngocho JS, Knippler ET, Chumba LN, Mmbaga BT, et

655	al. Retention in HIV Care During Pregnancy and the Postpartum Period in the

656	Option B+ Era. JAIDS J Acquir Immune Defic Syndr. 2018;77: 427–438.

657	doi:10.1097/QAI.0000000000001616

658	42.	Vrazo AC, Firth J, Amzel A, Sedillo R, Ryan J, Phelps BR. Interventions to

659	significantly improve service uptake and retention of HIV-positive pregnant

660	women and HIV-exposed infants along the prevention of mother-to-child

661	transmission continuum of care: systematic review. Trop Med Int Heal.

662	2018;23: 136–148. doi:10.1111/tmi.13014

663 43.	Ministry of Health and Child Care Zimbabwe (MoHCC). Operational and
664 Service Delivery Manual for the Prevention, Care and Treatment of HIV in
665 Zimbabwe. 2015;
666 44.	Willis N, Milanzi A, Mawodzeke M, Dziwa C, Armstrong A, Yekeye I, et al.
667 Effectiveness of community adolescent treatment supporters (CATS)
668 interventions in improving linkage and retention in care, adherence to ART
669 and psychosocial well-being: a randomised trial among adolescents living with
670 HIV in rural Zimbabwe. BMC Public Health. 2019;19: 117. doi:10.1186/s12889-

671	019-6447-4

672	45.	Brown LB, Getahun M, Ayieko J, Kwarisiima D, Owaraganise A, Atukunda M, et

673	al. Factors predictive of successful retention in care among HIV-infected men

674	in a universal test-and-treat setting in Uganda and Kenya: A mixed methods

675	analysis. Madiba S, editor. PLoS One. 2019;14: e0210126.

676	doi:10.1371/journal.pone.0210126

677	46.	Zürcher K, Mooser A, Anderegg N, Tymejczyk O, Couvillon MJ, Nash D, et al.

678	Outcomes of HIV-positive patients lost to follow-up in African treatment

679	programmes. Trop Med Int Heal. 2017;22: 375–387. doi:10.1111/tmi.12843

680

# 681 Supporting information

682 **S1 Figure:** Data management system for the National ART Program in Zimbabwe
683 **S1 Table**: Outcomes, follow time and time from testing to ART initiation for patients
684 who started antiretroviral therapy before and after the implementation of HIV “Treat
685 All” in the 9 pilots districts in Zimbabwe.
686 **S2 Table**: Attrition for patients who started antiretroviral therapy before and after the
687 implementation of HIV “Treat All” in the 9 pilots districts in Zimbabwe
688 **S1 File**: Retention and predictors of attrition among patients who started antiretroviral
689 therapy in Zimbabwe’s national antiretroviral therapy programme between 2012 and

690	2015. PLoS ONE 15(1): e0222309. https://doi.org/10.1371/journal.pone.022230

Figure	[Click here to access/download;Figure;Figure 1.tif](https://www.editorialmanager.com/pone/download.aspx?id=26947608&amp;guid=bccdf107-49a8-476e-9d8b-91d73f3f928c&amp;scheme=1)

Figure	[Click here to access/download;Figure;Figure 2.tif](https://www.editorialmanager.com/pone/download.aspx?id=26947609&amp;guid=a16bfda2-0441-4d7e-8b40-a475855b2c85&amp;scheme=1)

Figure

[Click here to access/download](https://www.editorialmanager.com/pone/download.aspx?id=26947610&amp;guid=a1afd85d-764e-4f73-9d9e-9bbd310fcbe7&amp;scheme=1)

[**Supporting Information** S1 Figure.tif](https://www.editorialmanager.com/pone/download.aspx?id=26947610&amp;guid=a1afd85d-764e-4f73-9d9e-9bbd310fcbe7&amp;scheme=1)

Supporting Information

[Click here to access/download](https://www.editorialmanager.com/pone/download.aspx?id=26947612&amp;guid=f5d88e4e-3f54-4235-b5c3-dcf533af1e7c&amp;scheme=1)

[**Supporting Information** S1 Table.docx](https://www.editorialmanager.com/pone/download.aspx?id=26947612&amp;guid=f5d88e4e-3f54-4235-b5c3-dcf533af1e7c&amp;scheme=1)

Supporting Information

[Click here to access/download](https://www.editorialmanager.com/pone/download.aspx?id=26947613&amp;guid=82386666-7aba-4edb-aa21-9572b10ba5df&amp;scheme=1)

[**Supporting Information** S2 Table.docx](https://www.editorialmanager.com/pone/download.aspx?id=26947613&amp;guid=82386666-7aba-4edb-aa21-9572b10ba5df&amp;scheme=1)

Supporting Information

[Click here to access/download](https://www.editorialmanager.com/pone/download.aspx?id=26947614&amp;guid=ec624d1e-ba50-42df-9efa-301d3060ea62&amp;scheme=1)

[**Supporting Information** S1 File.pdf](https://www.editorialmanager.com/pone/download.aspx?id=26947614&amp;guid=ec624d1e-ba50-42df-9efa-301d3060ea62&amp;scheme=1)
