# Supplementary material for: Patient-mix, programmatic characteristics, retention and predictors of attrition among patients starting antiretroviral therapy (ART) before and after the implementation of HIV “Treat All” in Zimbabwe
Source: PLoS One. 2020 Oct 19;15(10):e0240865. doi: 10.1371/journal.pone.0240865 (PMC7571688; doi:10.1371/journal.pone.0240865)
Supplement: Attachment — Submitted filename: Response to reviewers comments.docx [file pone.0240865.s007.docx]

**Reviewer #1:**

**The manuscript appears to be fairly sound with the authors having captured the critical data in line with the title. The language used is clear, easy to understand and unambiguous. The manuscript is informative and adds to the technical knowledge and understanding of subject area.**

- **The methods are sufficiently detailed.**
- **The study design is in sync with the subject of interest, and the study has sound statistical analyses.**
- **The results are well outlined with tables having correct titles**
- **The discussion section is very strong with relevant citations made**
- **The conclusions are solid and relating well with the findings.**
- **However, data is not accessible thus a limitation.**

RESPONSE: Many thanks for this positive feedback. We have submitted a de-identified dataset together with the resubmission.

**The authors should clarify the following sections to avoid confusion**

1. **In addition, the study setting information is not detailed enough for example, what percentage of clients do these nine districts serve?**

RESPONSE: We have expanded and added more detail in the study setting section and it now includes percentage of patients on ART, rural-urban distribution and extent of PEPFAR support. We added the following, “The nine districts had a total of 385 health facilities. During the pilot period, 121 (31.4%) of them were being supported by the United States Presidential Emergency Plan for AIDS Relief (PEPFAR) through its two main implementing partners: International Training Education and Centre for Health (I-TECH) and Organisation for Public Health Intervention Development (OPHID). Of the nine districts, Harare and Mutare are mainly urban while the rest are mainly rural with a few urban centres. By the end of 2018, the nine districts accounted for about 25% of patients on ART in the country”. *(Line 126-133)*

2. **In background, improve on the country and specific region context and literature review on the subject matter**

RESPONSE: We have added a paragraph on Zimbabwe HIV epidemiology and context in the background. The paragraph reads, “ Zimbabwe has a generalised epidemic with an estimated HIV prevalence of 13.7 % and 1.4 million PLHIV [1]. Of the estimated 1.4 million PLHIV, about 1.1 million are on ART. The country has progressed well towards meeting the 2020 UNAIDS 90-90-90 targets: 90% of PLHIV were aware of their HIV status; of that 86 % were on ART and among those on ART, 73% were virally suppressed [2]. The remarkable progress towards the 90-90-90 targets has been mainly due to the adoption of the WHO 2015 HIV guidelines, which included the “Treat All” recommendation and differentiated care. The guidelines were launched on World AIDS Day, 1st of December 2016 and currently “Treat All” is being implemented throughout the country. Prior to national scale-up, the “Treat All” recommendation was piloted in nine districts”. *(Line 87-96)*

3. **Under results, the authors were also assessing uptake of test and start among the different populations (men, adolescents) which is not related to the manuscript subject matter.**

RESPONSE: In our study we did not assess the uptake of test and start. We assessed the patient-mix before and after the implementation of “Treat All”. Patient-mix was defined as the diversity of patients in terms of age, sex, disease severity (WHO Stage, functional status), pregnancy and TB status at ART initiation *(Line 168-170).* We revised our title and objective and they now reads, “Patient-mix, programmatic characteristics, retention and predictors of attrition among patients starting antiretroviral therapy (ART) before and after the implementation of HIV “Treat All” in Zimbabwe” (title) *(Line 1-3)* and, “In the current study, we assessed the association between “Treat All” and patient-mix, programmatic characteristics, retention and predictors of attrition by comparing the ART cohorts enrolled during two periods before and after implementation of the “Treat All” policy” (objective) *(Line 112-115)*. We have included in the methods section, how we assessed the patient-mix using proportions. The section reads, “We assessed the patient-mix in terms age (children, adolescents and young adults, adults and elderly), sex (males females), disease severity (WHO Stage, functional status), pregnancy and TB status at ART initiation by calculating the proportion initiated on ART for each category among all those who were initiated on ART for the before and after “Treat All” cohorts by dividing the number initiated on ART per category with the total initiated in either cohort”. *(Line 195-200)*

4. **The age cut offs for the four categories i.e. children, adolescents and young adults, adults and elderly not clear**

RESPONSE: We have added the following statement in the methods clarifying the age categories, “The age was categorised into children: 0-14 years, adolescents and young adults: 15-24 years, adults: 25 – 49 years, and elderly: +50 years”. *(Line 170-172)*

5. **In the discussion, the authors aim to demonstrate low uptake of test and start among adolescents and increased uptake among men, however, the methods and data as shared does not fully support this**

RESPONSE: In our study, we assessed the patient-mix before and after “Treat All” and with the assumption that if a previously underserved subgroup is proportionally more frequent under “Treat All” this might imply that uptake has increased in this group. A more direct approach used to measure uptake relies on assessing the fraction of all HIV-infected individuals or the fraction of all ART-eligible individuals, who either initiated ART within a specified follow-up period from the date of HIV testing. We were unable to assess the fraction initiated within a specified period because ePMS does not collect individual-level data on those who do not start ART among all those tested for HIV. However, the ePMS has been updated to include an HIV testing module which collects individual-level testing and linkage to ART data. We highlighted this in the limitations of our study *(Line 450-459*) and adjusted our methods as described in 3.

6. **The authors introduced ART initiation in the discussion which was not in the study design nor population nor area of focus “We found that “Treat All” implementation did not increase the proportion of adolescents and young adults among those starting ART. To date, studies on whether HIV “Treat All” increases rapid ART initiation among adolescents are inconclusive”.**

RESPONSE: As described in 3 and 5 above, in our study, we assessed the patient-mix before and after “Treat All” and with the assumption that if a previously underserved subgroup is proportionally more frequent under “Treat All” this might imply that uptake has increased in this group.

**Reviewer #2:**

1. **The authors do not clearly state their aims or objectives in the manuscript. Thus it is difficult to follow through to assess claims or research questions being proposed by the authors as well as the findings that support them. The author should conclude their introduction section stating the aim or objective of the study.**

RESPONSE: We have adjusted the objective for the paper and moved it to the end of the introduction. We have revised the objective as follows, “In the current study, we assessed the association between “Treat All” and patient-mix, programmatic characteristics, retention and predictors of attrition by comparing the ART cohorts enrolled during two periods before and after implementation of the “Treat All” policy”. (*Line 112-115)*

2. **There are a lot of variables being assessed with no clear outcomes of interest.** **There were no definitions of variables or outcomes.**

RESPONSE: Our outcome variable was attrition, with as favourable outcome retention (being active in care at the end of the study period) and as composite adverse outcome the occurrence of death, LTFU, or stopping ART. *(Line 183-185)* We have defined patient-mix and programmatic exposure variables in the Methods (Study variables section) as follows; “Patient-mix was defined as the diversity of patients in terms of age, sex, disease severity (WHO Stage, functional status), pregnancy and TB status at ART initiation. The age was categorised into children: 0-14 years, adolescents and young adults: 15-24 years, adults: 25 – 49 years and elderly: +50 years. Functional status was categorised as either impaired for patients who were bedridden and ambulatory, and normal for those working. The TB status at ART initiation was classified as negative on screening, presumptive or on TB treatment. Programmatic characteristics included HIV testing modality, level of care, ART regimen and time from HIV testing to ART initiation. Time from testing to ART initiation was defined as the time between the date of HIV testing and date ART initiation. *(Line 168 – 178)*

3. **The structure of most of the sentences needs re-phrasing for a clear understanding.**

RESPONSE: The sentences have been rephrased and are shown in track changes.

4. **The methods section is not well written and needs to be detailed but clear and concise. The study procedure needs to be written with more details so the study can be replicated elsewhere by following the detailed study procedure description by authors. The methods section in the current state that it is does not allow other investigators elsewhere to follow and fully replicate the study.**

RESPONSE: We have revised the methods sections. Firstly, we have revised the Data sources and study procedures section to clearly illustrate how we collected the data for the study and it now reads, “We used routinely collected National ART Program data in our study. Every patient within the program has a manual paper-based medical record, i.e. Patient ART/Opportunistic Infection Booklet. Patients medical records (baseline and routine visits) are first collected in this paper-based medical record. The data are then entered into the ePMS by either health care workers or data entry clerks available at some of the health facilities. Every month health facilities submit an encrypted back-up of the ePMS data to the district-level where all data are consolidated. The consolidated district-level data are then submitted to the national level, where it is further consolidated into one national database happens. The MoHCC, together with supporting partners, have put in place strategies to improve ePMS data quality and facility reporting rates. The strategies include regular support and supervision visits and on-site data verification. The ePMS database has also been designed with inbuilt check systems to improve data quality. Data for this study were extracted from the national database in an MS Excel format and exported to Stata were it was cleaned (synchronised and deduplicated) before analysis (S1 Figure)”. *(Line 149-164)*

Secondly, we revised the Study variables section to include the definition of variables, “Patient-mix was defined as the diversity of patients in terms of age, sex, disease severity (WHO Stage, functional status), pregnancy and TB status at ART initiation. The age was categorised into children: 0-14 years, adolescents and young adults: 15-24 years, adults: 25 – 49 years and elderly: +50 years. Functional status was categorised as either impaired for patients who were bedridden and ambulatory, and normal for those working. The TB status at ART initiation was classified as negative on screening, presumptive or on TB treatment. Programmatic characteristics included HIV testing modality, level of care, ART regimen and time from HIV testing to ART initiation. Time from testing to ART initiation was defined as the time between the date of HIV testing and date ART initiation. *(Line 168 – 178)*

Lastly we added the following in the data analysis, “We assessed the patient-mix by age (children, adolescents and young adults, adults and elderly), sex (males females), disease severity (WHO Stage, functional status), pregnancy and TB status at ART initiation by calculating the proportion initiated on ART for each category among all those who were initiated on ART for the before and after “Treat All” cohorts by dividing the number initiated on ART per category and the total initiated in either cohort. We assessed the change in programmatic characteristics (HIV testing modality, level of care, ART regimen and time from HIV testing to ART initiation) by calculating the proportion initiated on ART for each category among all those who were initiated on ART for the before and after “Treat All” cohorts by dividing the number initiated on ART per category and the total initiated in either cohort. Time from testing to ART initiation was calculated by subtracting the date when the patient was initiated on ART with the date of HIV testing”. *(Line 195-207)*

“Data on weight, height, CD4 and viral load were more than 50% missing and were excluded from the study. Functional status, WHO Stage, TB status at ART initiation and HIV testing modality were less than 30% missing and were included in the study after correcting for the missingness through multiple imputation”. *(Line 216-219)*

**Most comments can also be found in the attachment that has been uploaded**

We have extracted the following comments which were inserted via track changes in the manuscript and responded to them below.

5. **There is no literature reference or statement on the disease epidemiology, existing programme characteristics, ART services in Zimbabwe for contextual purposes. Authors should please add that information**

RESPONSE: We have added a paragraph on the context and HIV epidemiology in Zimbabwe in the background. *(Line 87-96)*

6. **Sites selected not representative as only 9 out of 63 sites were used. Why were they selected? Is it because they are the high burden sites? Authors indicate they were pilot sites in 2016. How does that relate to the study objectives now reason for selection? From the narrative above, Zimbabwe started treat all policy implementation in 2016 and has scaled up. Using programmatic data, why did authors use only 9 out of 63 districts. These nine districts are also sites supported by PEPFAR. Resource allocation for HIV services in these districts may differ from other districts with little or support from other organizations. Unless the aim was to look at characteristics in PEPFAR supported sites.**

RESPONSE: We decided to use the nine pilot districts to assess the effect of “Treat All” on retention because these were the first districts to implement “Treat All” in Zimbabwe. These were the only districts that piloted the “Treat All” recommendation and we purposively included all of them. The selection was not based on burden but by end of 2018 they accounted for 25% of the number of patients on ART in Zimbabwe. The 9 districts were selected from a total of 63, and they were from 4 of the 10 provinces. Of the nine districts, Harare and Mutare are mainly urban while the rest are mainly rural with a few urban centres *(Line 125-133*). However, since our study was based on health facilities with ePMS, which is only available in high volume health facilities, we have highlighted that this selection may hamper the generalisability of our findings in the discussion. *(Line 441-443)*

We chose the pilot districts because they have the oldest “Treat All” cohorts in the country, thus also allowing you to assess attrition during a follow-up period of at least 12 months. We wanted to get the outcomes based on the early on the experiences of the pioneer districts. Since the scale-up of “Treat All”, there has been a lot of changes on how HIV “Treat All” is now being implemented based on the identified gaps. This therefore, means studying “Treat All” using programme data years into implementation might not give the true picture. During the period of the pilot only 31.4% of the health facilities in all the 9 pilots districts were being supported directly by PEPFAR and of 72 health facilities included in our study, 16 of them were not supported by PEPFAR, hence the fact that our study aimed at assessing PEPFAR supported sites is not correct. We included partner support i.e. PEPFAR as an exposure variable in our analysis but was not associated with improvement in attrition. This might have been because early on in “Treat All” implementation the attention was on HIV testing and ART initiation with little focus on retention in care. However, PEPFAR has since started supporting all facilities within a district they are operating in since their Country Operation Plan of 2018.

7. **What is meant by updated data? This is because your data analysis section caters for missing data so am wondering why the rest of the facilities (59) were left out.**

RESPONSE: We replaced updated with submitted.*(Line 141)* On a monthly basis, facilities submit ePMS data to the district for onward submitted to the national level. However, due to logistical challenges some facilities do not submit. We only included facilities with submission up to date at national level. What we addressed with multiple imputation was the missingness of the variables

8. **Study sample not properly defined here. Aside the months/periods stated, who are the specific population being dealt with and their common characteristics. What were the inclusion and exclusion criteria?**

RESPONSE: We have adjusted and revised our the methods sections and it now reads, “We included also patients who were initiated on first-line ART during the before and after Treat All periods, and the common criteria being patients starting first-line ART. We included children, adolescents, adults, elderly and pregnant women. Patients who were restarted on ART after stopping were excluded”. *(Line 143-147)* The other common criteria are shown in Table 1 and Table 2.

9. **These tells us the data source is ePMS. The authors should add how the data were extracted, the tool(s) used, how data validation was ensured and how privacy or confidentiality was ensured.**

RESPONSE: No tools were used for data extraction. The data were extracted from the ePMS database in Excel format based on our list of variables and outcomes. In terms of data validation, the MoHCC together with partners have put in place strategies to improve the data quality and facility reporting rates. The strategies include regular support and supervision visits and onsite data verification. The ePMS database has also been designed with in built check systems to improve the data quality *(Line 149-164).*

Confidentiality and privacy was ensured by removing the district, province, health facility names and the patient unique identification number. *(Line 243-245)*

10. **What was the outcomes in patients initiated on same day with regards to retention in care?**

RESPONSE: We did not assess the effect of same day ART initiation on retention in our study. The primary aim of our study was to assess retention between before and after “Treat All”. Same day ART initiation started to be implemented as part of “Treat All” and was barely implemented prior. “Treat All” is implemented as package which includes HIV testing, linkage to care, clinical and psychological readiness assessment, retesting before ART initiation, same-day ART initiation, follow-up and retention in care. Because of that difference we were unable to include same day ART initiation as a variable in our study but have recommended further research on the effect of same day using a cohort which started ART only under “Treat All”.

11. **“This collided with findings from our previous evaluation that patients starting ART at a higher CD4 cut-off might be at risk of attrition. Patients started on ART under “Treat All”, possibly have low-risk perception due to the absence of symptoms and might be less motivated to adhere to lifelong daily ART”. This explanation is contrary to your finding of worsening or advance disease being also associated with lower retention in care or increasing risk of attrition. Kindly justify this.**

RESPONSE: With the implementation of Treat All patients are now being started on ART early i.e. regardless of CD4 or immunological criteria. Though patients are being started on ART early, not all the patients are presenting at ART initiation with a CD4 count higher than 200. According to programme data, about 30% of patients who started on ART in 2019 presented with advanced HIV disease i.e. CD4 <200 or WHO Stage 3 & 4. So the cohort starting ART under Treat All is not uniform but consists of group with advanced disease. Those with advanced disease will be lost mainly due to mortality and morbidity [3–5]. while those with higher CD4 will lost due due to low risk perception. [6,7] .

12. **“The study was the first to assess the performance of the ART programme since the start of HIV “Treat All” policy implementation and allowed us to test and confirm findings from our previous evaluation”, What has been tested is not clear from your previous study. The authors should outline this clearly under the methods section.**

RESPONSE: We have deleted “to test and” and left confirm since it was more of confirming findings from our previous evaluation. We have included the key findings of our previous evaluation in the background *(Line 107 – 112)* and we refer and compare these findings in the discussion.

13. **“Most of the attrition in our study was explained by LTFU. Some of these patients reported as LTFU might be still alive and on ART (unreported self-transfers), stopped ART or unreported deaths. Because of missing data, we were also unable to compare the viral load suppression rates between the before and after HIV “Treat All” cohorts. Unfortunately, it was impossible to solve this by merging ePMS and laboratory data, due to missing or inconsistent format of the unique patient identifier between the various testing laboratories. Strategies to improve data quality and making sure the existing electronic data collection systems are interoperable need urgent attention.”**

**I don’t think these statements are limitations**

RESPONSE: The fact that most of attrition was account for by LTFU is limitation because studies have shown that most of patients declared LTFU are not truly LTFU (unreported deaths and silent self-transfers) [8] . This therefore means we might have over- or under-estimated attrition. Among the unknown real-world effects of Treat All is viral load suppression. In our study we were unable to assess virological outcomes because viral load data was missing in the ePMS.

14. **“From our field experience, we have noticed the implementation of “Treat All” encompasses many aspects across the cascade of care. The package includes innovative HIV testing and linkage to care strategies, clinical and psychological readiness assessment, retesting before ART initiation, same- day ART initiation, follow- up and retention in care strategies. On top of the HIV cascade of care issues; there are also health systems challenges anticipated from the abrupt increase in the number of patients now being followed-up in care”**

**“Men continue to be at risk under “Treat All”; further research is needed on how ART delivery can be streamlined to retain them in care. Most studies, have focused on patient-level factors on ART outcomes. This, therefore, calls for studies looking into the impact of health facility- level characteristics on ART outcomes since performance might vary across facilities”**

**These statements are not needed here**

RESPONSE: The statements are supporting the need to study separately the components of “Treat All” and health-facility level factors on retention and follow-up on why we found men at risk of attrition. We have rephrased and kept the statements and they now reads as follows;

“We evaluated the effect of “Treat All” as one intervention but “Treat All” is being implemented as a package with several components across the cascade care (HIV testing, linkage to care, clinical and psychological readiness assessment, retesting before ART initiation, same-day ART initiation, follow-up and retention). Our study was unable to unpack these components and assess their individual effects on retention in care”. *(Line 481-490)*

“Men continue to be at risk of attrition under “Treat All”; further research is needed on how ART delivery can be streamlined to retain them in care. Most studies, including ours, have focused on patient-level factors on ART outcomes. Lumping patients from different facilities together may cause loss of information on specific health facility-level factors that influence retention. This, therefore, calls for studies looking into the impact of health facility- level characteristics on ART outcomes since performance might vary across facilities”. *(Line 491-497)*

**References**

1.	Ministry of Health and Child Care (MoHCC). 2019 National HIV Estimates. 2019.

2.	UN Joint Programme on HIV/AIDS (UNAIDS). Global AIDS Update. 2020. https://www.unaids.org/sites/default/files/media_asset/2020_global-aids-report_en.pdf.

3.	Harries AD, Zachariah R, Lawn SD, Rosen S. Strategies to improve patient retention on antiretroviral therapy in sub-Saharan Africa. Trop Med Int Heal. 2010;15(SUPPL. 1):70–75.

4.	Lawn SD, Harries AD, Wood R. Strategies to reduce early morbidity and mortality in adults receiving antiretroviral therapy in resource-limited settings. Curr Opin HIV AIDS. 2010;5(1):18–26.

5.	Marazzi MC, Liotta G, Germano P, Guidotti G, Altan AD, Ceffa S, et al. Excessive Early Mortality in the First Year of Treatment in HIV Type 1-Infected Patients Initiating Antiretroviral Therapy in Resource-Limited Settings. AIDS Res Hum Retroviruses. 2008;24(4):555–560.

6.	Stafford KA, Odafe SF, Lo J, Ibrahim R, Ehoche A, Niyang M, et al. Evaluation of the clinical outcomes of the Test and Treat strategy to implement Treat All in Nigeria: Results from the Nigeria Multi-Center ART Study. PLoS One. 2019;14(7):e0218555.

7.	Charurat M, Oyegunle M, Benjamin R, Habib A, Eze E, Ele P, et al. Patient Retention and Adherence to Antiretrovirals in a Large Antiretroviral Therapy Program in Nigeria: A Longitudinal Analysis for Risk Factors. PLoS One. 2010;5(5):e10584.

8.	Zürcher K, Mooser A, Anderegg N, Tymejczyk O, Couvillon MJ, Nash D, et al. Outcomes of HIV-positive patients lost to follow-up in African treatment programmes. Trop Med Int Heal. 2017;22(4):375–387.
